# Supplementary material for: Implementation fidelity of tuberculosis preventive therapy for under five children exposed to sputum smear positive pulmonary tuberculosis in Kaski district, Nepal: An implementation research
Source: PLoS One. 2022 Feb 16;17(2):e0263967. doi: 10.1371/journal.pone.0263967 (PMC8849459; doi:10.1371/journal.pone.0263967)
Supplement: S1 Table — (DOCX) [file pone.0263967.s001.docx]

**Socio-demographic characteristics of the participants involved in qualitative study**

| **Character** | **Categories** | **In depth**  **Interview (n=16)** | **FGD**  **(n=1)** | **Total** |
| --- | --- | --- | --- | --- |
| Gender | Male | 5 | 0 | 5 |
|  | Female | 11 | 8 | 19 |
| Total |  | 16 | 8 | 24 |
| Age | <40 | 13 | 4 | 17 |
|  | >40 | 3 | 4 | 7 |
| Education | Elementary | 1 | 1 | 2 |
|  | Secondary/Technical health  training | 5 | 5 | 10 |
|  | Intermediate level | 6 | 2 | 8 |
|  | Bachelor and above | 4 | 0 | 4 |
| Participants | Health care provider | 6 | 0 | 6 |
|  | TB focal person | 1 | 0 | 1 |
|  | SR representative | 1 | 0 | 1 |
|  | Parents/caretaker (Initiated  children) | 4 | 0 | 4 |
|  | Parents/caretaker (Not  Initiated children) | 4 | 0 | 4 |
|  | Contact tracers | 0 | 8 | 8 |
