# Supplementary material for: Implementation fidelity of tuberculosis preventive therapy for under five children exposed to sputum smear positive pulmonary tuberculosis in Kaski district, Nepal: An implementation research
Source: PLoS One. 2022 Feb 16;17(2):e0263967. doi: 10.1371/journal.pone.0263967 (PMC8849459; doi:10.1371/journal.pone.0263967)
Supplement: S1 File — (DOCX) [file pone.0263967.s002.docx]

**Secondary data extraction sheet**

| **Data extraction from TB register** | | | | | | | | | |
| --- | --- | --- | --- | --- | --- | --- | --- | --- | --- |
| **District:** | | | | | | | | | |
| **Fiscal Year:** | | | | | | | | | |
| SN | TB Patients characteristics | | | | | | | TB characteristics | |
|  | Name | Date of registration | Age | Sex | Ethnicity Code | HIV status | Documentation of phone number | Type | Treatment |
|  |  |  |  |  |  |  |  |  |  |
|  |  |  |  |  |  |  |  |  |  |
|  |  |  |  |  |  |  |  |  |  |
|  |  |  |  |  |  |  |  |  |  |
|  |  |  |  |  |  |  |  |  |  |
|  |  |  |  |  |  |  |  |  |  |

**Secondary data extraction sheet**

| **Data extraction from Contact tracing register** | | | | | | | | | **Data extraction from TBPT register** | | | | | | |
| --- | --- | --- | --- | --- | --- | --- | --- | --- | --- | --- | --- | --- | --- | --- | --- |
| **Fiscal Year** | | | | | | | | | | | | | | | |
| **District** | | | | | | | | | | | | | | | |
| S.N | Name | Contact traced Status | Date of contact tracing | Total number of contact | Documentation of age of contact person | Total Number of under 5 years children | Total number of 5-15 years children | Adults  Above 15 years | TBPT enrollment status | Date of enrollment | Age | Sex | Relation with index case | Education of parents | Therapy status |
|  |  |  |  |  |  |  |  |  |  |  |  |  |  |  |  |
|  |  |  |  |  |  |  |  |  |  |  |  |  |  |  |  |
|  |  |  |  |  |  |  |  |  |  |  |  |  |  |  |  |
|  |  |  |  |  |  |  |  |  |  |  |  |  |  |  |  |
