# Supplementary material for: Implementation fidelity of tuberculosis preventive therapy for under five children exposed to sputum smear positive pulmonary tuberculosis in Kaski district, Nepal: An implementation research
Source: PLoS One. 2022 Feb 16;17(2):e0263967. doi: 10.1371/journal.pone.0263967 (PMC8849459; doi:10.1371/journal.pone.0263967)
Supplement: S2 File — (DOCX) [file pone.0263967.s003.docx]

**Key Informant Interview and In- depth Interview Guide**

**Questionnaire for In-depth Interview among Health care provider**

1. How do you counsel SS+ patient? What are the information

(probe : Household contact, under five years contact, TBPT)

1. Do you always ask SS+ patients if they have under five children in their home? Do you ask by yourself or hand over to contact tracer to visit home and trace?
2. What are you going to do if the SS+ patients refer that there are children five years living with them?

( Refer to HF for further treatment, screen for TB)

1. What thing do you consider while screening for TB?

(TB screening questionnaire, 4 sign and symptoms)

1. What will you if they don’t have TB?

(Enroll in TBPT)

1. What is your understanding on TBPT

(Advantage, disadvantage, treatment procedure)

1. How do you counsel before enrolling in therapy?

(side effect, Dose, duration, sign and symptoms of negative reaction)

1. For how long do you give medicine?
2. How do you find TBPT guideline?

((prob: clarity, simple, complex, rolling out eligible cases)

1. How do you ensure that children are taking medicine which you have given?

(probe : homevisit, how often)

1. Is it easy/difficult to do home visit? Why easy/ why difficult?

(probe: insufficient manpower, overload, insufficient time)

1. Do you give necessary information to contact tracer for contact tracing? With in how much time contact tracer visit patient home?
2. Do you properly make report data which contact tracer bring from community? What are barrier in recording and reporting?
3. Have you ever take training regarding this program? Do you think training is important? How long was the discussion session? Do you think that was enough?
4. How many health worker and contact are involve in this program. Is that enough? Do you think contact tracer are loyal to the program?
5. How is the collaboration between contact tracer, DPHO and SR staff?
6. What is your particular feelings and emotion about this program IPT?
7. What are the distracting factor in providing IPT services?

(pro: overload tiredness, drug supply, diagnostic facility, )

1. Are you getting your incentives regularly? Do you think, these kind of reward encourage you for better performances?

(probe sufficient, not sufficient)

1. Government is giving travel cost for children enrol in therapy? Do you think this encourage them to enrol in therapy. Is it important for patient. Or they enroll because they are aware?
2. According to you, what should be improved from the provision of IPT for under 5 years program in this PHC?
3. Do you want add something more?

**In-depth Interview Questionnaire for Parents/caretaker whose children are not enroll in TBPT**

1. Have you ever heard about IPT? IF yes, by whom ? What is your understanding about IPT?

(probe: worthwhile, strength, weakness)

1. Did contact tracer, health provider counsel you about IPT? What did they say?

(Probe: drug regimen, duration, where to get, how to take)

1. How often they visit your home?
2. Why didn’t you start IPT to your children?

(pro: distance to health facility, child don’t like taste of medicine, relation with index cases ,fear of side effect)

1. What do you think about travel cost that government has been providing for children enrolling in IPT?
2. What is your family/husband perception about IPT? (pro support)
3. Have you ever communicate with parents/caretaker who do not enroll their children in IPT ? What are their suggestion?
4. Will you enroll in therapy if medicine is given community. What is your preference ?
5. Do you have anything to add?

**In-depth Interview Questionnaire for Parents/caretaker whose children completed /under therapy**

Questions

1. Have you ever heard about IPT? IF yes, by whom ? What is your understanding about IPT? (probe: worthwhile, strength, weakness)
2. How is your experience when bringing your children to the services?
3. Why do you start and complete IPT to your children?
4. What do you think about travel cost that government has been providing for children enrolling in IPT? Did it motivate you to take services?
5. What is your family/husband perception about IPT?
6. Have you ever communicate with parents/caretaker who do not enroll their children in IPT ? What are their suggestion?
7. Did contact tracer, health provider counsel you about IPT? How often they visit your home?
8. Are you satisfy with their service? (Probe: Counselling, follow up)
9. What would you suggest to improve quality of service?
10. Do you have any additional comment about IPT?

**Focus Group discussion Guideline with contact tracer**

1. What is your understanding about contact tracing? Do you contact screen all SS+ patient?
2. How do address issue of privacy and confidentiality during contact screening?
3. How fast do you do contact screening once SS+ case is registered in Health facility?

(problem, particular experience, early and late date)

1. Have you ever heard about tuberculosis preventive therapy? What is your understanding?

( Probe: Pros and cons, treatment regimen)

1. Have you ever enroll or refer under five years children for TBPT from your own effort?
2. Do you have any eligible children for preventive therapy but not enroll in therapy in your community ?

(Probe: delay in contact tracing, lack of message dissemination)

1. Have you had any training for this program? Do you think training is important?
2. How is the support from health care provider ?
3. (probe: regularly providing detail of SS+ pulmonary Tb cases)
4. How do you find guidelines and SOPs regarding IPT?
5. (prob: clarity, rolling out eligible cases)
6. What are the distracting factor in IPT services?
7. (probe: convincing parents, )
8. Are you getting your incentives regularly? Do you think, these kind of reward encourage you for better performances?
9. What is your particular feelings and emotion about IPT?
10. What are the additional challenges/ you are facing?
